# Women's Knowledge, Attitudes, and Practice About Female Genital Cosmetic Surgery: A Cross-Sectional Study in Saudi Arabia

**DOI:** 10.7759/cureus.49201

**Published:** 2023-11-21

**Authors:** Hatim Alrashed, Norah H Alsultan, May H AlQahtani, Reem F Bahakeem, Dorar Alharbi, Norah Alabdullatif, Lubna Aloufi, Enas Z Bedaiwi, Albagir M Hassan

**Affiliations:** 1 Medicine and Surgery, College of Medicine, Jouf University, Aljouf, SAU; 2 Medicine, King Faisal University, Hasa, SAU; 3 Medicine, Princess Norah Bint Abdulrahman University, Riyadh, SAU; 4 Faculty of Medicine, Umm Al-Qura University, Makkah, SAU; 5 Medicine, Taibah University, Medina, SAU; 6 College of Medicine, Qassim University, Al-Qassim, SAU; 7 College of Medicine, King Saud University, Riyadh, SAU; 8 Obstetrics and Gynaecology, Umm Al-Qura University, Makkah, SAU

**Keywords:** saudi arabia, women, practice, attitude, knowledge, aesthetic vaginal surgery, cosmetic genital surgery

## Abstract

Introduction

Vulvoplasty, or female genital cosmetic surgery (FGCS), refers to any surgical alteration of the vaginal or labial anatomy for aesthetic or medical reasons. It aims to restore or enhance the female genitals and can involve multiple procedures such as labiaplasty, clitoral unhooding, monsplasty, vaginoplasty, hymenoplasty, G-spot augmentation, frenuloplasty, perineoplasty, fat injections, or a combination of these. Labiaplasty is currently the most popular procedure among patients. Since the public is exploring FGCS benefits in the media, it can be foreseen that it will soon gain popularity among the population of Saudi Arabia. As a result, the purpose of this study is to assess women's attitudes and knowledge towards genital cosmetic surgeries in Saudi Arabia.

Methods

This correlational cross-sectional survey was conducted in Saudi Arabia among women aged 18 years and above, using an online self-structured questionnaire distributed from April 2023 to October 2023. The sample size of 594 respondents was determined based on a minimum requirement of 500 participants, with a confidence level of 95% and a study power of 95%. A convenient sampling method was employed to select participants, and data collection was carried out through a self-administered online questionnaire distributed via various social media platforms. The survey was self-structured, and Cronbach's alpha values for all sections were greater than 0.7. These sections include demographic characteristics, sexual life and obstetric history, and women's knowledge, practices, and attitudes toward FGCS. Descriptive analysis, chi-square test, and t-test were used for data analysis using SPSS software.

Results

A total of 589 eligible women were included in the study; 284 (48.2%) were from the central region, and ages ranged from 18 to 65 years, with a mean age of 33.5 years. A total of 401 (68.1%) were married, 366 (62.1%) had a bachelor degree. Two hundred and ninety-one (49.4%) participants heard about FGCS, 165 (28%) knew that it involves surgical procedures to change the appearance of the female genitalia, 144 (24.4%) said it is also known as vaginal rejuvenation or designer vagina surgery and 200 (34%) knew it can involve procedures such as labiaplasty, clitoral hood reduction, or vaginal tightening, while 190 (32.3%) reported it is sometimes done for aesthetic reasons but may also be done for medical reasons. Only 45 (7.6%) had undergone FGCS, but 112 (19%) confirmed they consider undergoing FGCS for themselves. Four hundred and ninety-eight (84.6%) participants thought that it's important to have access to support services, such as counseling or peer support, 471 (80%) expressed that it's important that healthcare providers in Saudi Arabia are knowledgeable about FGCS, 425 (72.2%) were concerned about the potential risks and complications of FGCS.

Conclusion

In conclusion, the current study revealed that nearly one out of five women were knowledgeable about FGCS, mainly about the nature and types of the procedure. Higher knowledge levels about FGCS were associated with younger age, higher educational levels, and women who were more likely to consider undergoing FGCS in the future.

## Introduction

Some women choose to undergo non-medical cosmetic surgery to alter the appearance and structure of their external and internal genitalia. This is known as female genital cosmetic surgery (FGCS) and includes procedures such as vaginoplasty, labiaplasty, hymenoplasty, mons pubis liposuction, G-spot augmentation, vaginal rejuvenation or laser rejuvenation, and orgasm-shot [1,2]. Labiaplasty is the most frequently performed procedure [3]. For women concerned about the appearance of their genitalia, FGCS provides significant psychological advantages, and following FGCS, the majority of women with severe deformity reported less pain or discomfort during everyday activities and sexual activity [4]. It has been reported by the American Society for Aesthetic Plastic Surgery that there is a growing demand for FGCS in the United States. Specifically, there has been a 23.2% increase in labiaplasty surgeries between 2015 and 2016. Similarly, Australia saw a threefold increase in labiaplasties between 2003 and 2013, while the UK experienced a fivefold increase [5]. Social media certainly contributed the most to this rise in awareness; Crockett et al. conducted a study on the influence of plastic surgery, and they discovered that television shows influenced four out of five patients to seek cosmetic surgery [6]. All the studies that were conducted in Saudi Arabia targeted the patients' satisfaction, the perspective of their experience after the operation, and their motives [7]. However, several studies evaluated physicians' knowledge from different subspecialties, and the reported knowledge level was suboptimal, but their attitudes improved [8,9]. Since FGCS benefits are being explored by the public, it can be foreseen that it will gain popularity in the near future among our population. The rise in popularity of cosmetic surgery is likely due to media influence. In a study conducted by Crockett et al. on the impact of plastic surgery, 80% of patients reported being influenced by television shows to pursue a cosmetic procedure [6]. It's also worth noting the significant expectations that often follow such surgeries [10,11]. The current study aimed to discover the knowledge and attitudes of Saudi women about female genital cosmetic surgery.

## Materials and methods

A correlational cross-sectional study was conducted based on an online structured questionnaire distributed to Saudi women aged 18 years or more. The study was conducted from April 2023 to October 2023. The inclusion criteria comprised adult women aged 18 years and above residing in any region of Saudi Arabia. On the other hand, women aged less than 18 years of age, females who refused to participate, and those with incomplete survey answers were excluded. The sample size was determined using the G-power analysis program, which recommended a minimum of 500 participants for a study with a 95% confidence level and a study power of 95%. For this study, a total of 594 respondents were included. 

To select participants, a convenient sampling technique was employed. The data collection process involved the use of a self-structured survey. The validity of the questions was assessed, and Cronbach's alpha values for all sections were found to be satisfactory, being greater than 0.7.

The questionnaire was presented in Google Forms and distributed through various social media platforms. Multiple data collectors were involved until no further responses were received. 

The consent was obtained from the participants first. After that, answering an online questionnaire was requested. The questionnaire was designed in Arabic and contained three sections. The first section collected the bio-demographic data. The second section covered the sexual, gynecological, and obstetrical information regarding the recent experience of pregnancy and delivery, history of infections, and genital examination performance and frequency. The third section contained women's knowledge about FGCS, their source of information, and their need for more information. In addition, the questionnaire collected data about women's practice of FGCS, types of undergone cosmetic surgeries (FGCS or other cosmetic surgeries), and their attitude toward FGCS and the physician's role. Data was collected, coded, and subjected to direct analysis with the aim of minimizing bias. 

Data analysis

The data were collected, reviewed, and then entered in Statistical Package for Social Sciences version 21 (SPSS; IMB Inc., Armonk). All statistical methods used were two-tailed with an alpha level of 0.05, considering a p-value less than or equal to 0.05 to be significant. Overall knowledge level regarding FGCS was assessed by summing up discrete scores for different correct awareness items. The overall knowledge score was categorized as a poor level if the participants' score was less than 60% of the overall score and a good level of awareness if the participants' score was 60% or more of the overall score. Descriptive analysis was done by presenting frequency distribution and percentages for study variables, including participants' personal data, medical, sexual, and obstetric history. Also, the frequency distribution of the women's knowledge about FGCS, their practice, attitudes, and overall knowledge level were tabulated. Cross tabulation was applied to show factors associated with participants' knowledge about genital cosmetic surgery using the Pearson chi-square test and exact probability test if there were small frequency distributions.

## Results

A total of 589 eligible women were included, 284 (48.2%) of them from the central region. Ages ranged from 18 to 65 years; 38.7% (228 ) comprised the largest group aged 25-34. A total of 401 (68.1%) were married, 366 (62.1%) had bachelor's degree. Two hundred and fifty-seven (43.6%) participants were housewives, 176 (29.9%) were employed full-time, 39 (6.6%) worked part-time and 24 (4.1%) were self-employed. A monthly income of less than 1333 USD (<5000 SR) was reported among 331 (65.2%) women, and 130 (22.1%) had a monthly income of 1333-2666 USD (5000-10000 SR). A total of 57 (9.7%) were identified as having diabetes mellitus, 43 participants (7.3%) had hypertension, and 56 participants (9.5%) were diagnosed with hyperthyroidism. The majority of the participants, 424 participants (72%), reported no significant health problems (Table 1).

**Table 1:** Bio-demographic data of study females in Saudi Arabia

Demographic data	No	%
Region		
Central region	284	48.2%
Northern region	33	5.6%
Eastern region	61	10.4%
Western region	165	28.0%
Southern region	46	7.8%
Age in years		
18-24	152	25.8%
25-34	228	38.7%
35-44	124	21.1%
45-54	69	11.7%
55+	16	2.7%
Marital status		
Single	143	24.3%
Married	401	68.1%
Divorced / widow	45	7.6%
Educational level		
Below secondary	23	3.9%
Secondary / diploma	148	25.1%
Bachelor degree	366	62.1%
Post-graduate	52	8.8%
Employment		
Housewife	257	43.6%
Student	93	15.8%
Employed full-time	176	29.9%
Employed part-time	39	6.6%
Self-employed	24	4.1%
Monthly income		
<5000 SR	331	56.2%
5000-10000 SR	130	22.1%
10000-15000 SR	74	12.6%
15000-20000 SR	34	5.8%
>20000 SR	20	3.4%
Medical diseases		
None	424	72.0%
Diabetes	57	9.7%
Hypertension	43	7.3%
Hypothyroidism	56	9.5%
Cardiac disease	15	2.5%
Asthma	47	8.0%

Sexual and obstetric history of study participants are presented in Table 2. As for sexual history, 276 (46.9%) of married women reported having sex for the first time at the age of 18-24 years. One hundred and eighty-seven (41.9%) married women had sexual activity 1-2 times a week, and 131 (29.4%) had sexual activity less than once a week. Only 38 (6.5%) had sexually transmitted infections. Concerning obstetric history, most of the married women (80%) got pregnant, 240 (70%) had a normal vaginal delivery, of whom 81 (22.7%) did not experience any complications during pregnancy or delivery. Perineal tear/episiotomy during delivery was reported among 142 (41.4%) women who gave birth. Out of the total participants, 286 (48.6%) underwent a gynecological examination, primarily in response to specific concerns or issues, with the majority of 228 (79.7%) seeking such examinations only when faced with health concerns. Among the study participants, regular self-examinations of the genitalia area were reported by 168 women (28.5%), with the majority of them, 104 (62.3%), indicating that they performed such examinations only in response to specific concerns or health issues. 

**Table 2:** Sexual, obstetrical and gynecological history of the participants NVD - normal vaginal delivery; CS - Cesarean section

Sexual, obstetrical and gynecological history		No	%
Age at first sexual intercourse	Never had sexual intercourse	143	24.3%
	<18	45	7.6%
	18-24	276	46.9%
	25-34	69	11.7%
	35+	56	9.5%
Frequency of sexual activity per week	Less than once a week	131	29.4%
	1-2 times a week	187	41.9%
	3-4 times a week	93	20.9%
	> 4 times a week	35	7.8%
History of sexually transmitted infections (STIs)	Yes	38	6.5%
	No	551	93.6%
Have you ever been pregnant?	Yes	357	80.0%
	No	89	20.0%
How many children have you given birth to?	None	14	3.9%
	1-4	264	73.9%
	5+	79	22.1%
Mode of delivery	NVD	240	70.0%
	CS	83	24.2%
	Instrumental delivery	20	5.8%
Have you experienced any complications during pregnancy or delivery?	Yes	81	22.7%
	No	276	77.3%
Have you undergone perineal tear/episiotomy during the previous delivery?	Yes	142	41.4%
	No	172	50.1%
	Not sure	29	8.5%
Have you ever had a gynecological exam?	Yes	286	48.6%
	No	303	51.4%
If yes, how often do you have gynecological exams?	Annually	38	13.3%
	Every 2-3 years	20	7.0%
	Only when there is a problem	228	79.7%
Do you perform regular self-examinations of your genitalia?	Yes	168	28.5%
	No	421	71.5%
If yes, how often do you perform self-examinations?	Monthly	23	13.8%
	Every 3-6 months	40	24.0%
	Only when there is a problem	104	62.3%
Have you ever undergone any gynecological procedures or surgeries?	Yes	48	8.1%
	No	541	91.9%

Women's knowledge about genital cosmetic surgery in Saudi Arabia is presented in Table 3. Two hundred and ninety-one 291 (49.4%) participants heard of female genital cosmetic surgery (FGCS), 165 (28%) said it involves surgical procedures to change the appearance of the female genitalia, 144 (24.4%) told it also known as vaginal rejuvenation or designer vagina surgery and 200 (34%) knew it could involve procedures such as labiaplasty, clitoral hood reduction, or vaginal tightening, while 190 (32.3%) reported it is sometimes done for aesthetic reasons but may also be done for medical reasons. A total of 316 (53.7%) participants expressed a desire to acquire further knowledge about FGCS, while 105 (17.8%) believed that there is a sufficient amount of information available about FGCS in Saudi Arabia. Only 131 participants (22.2%) reported that information or societal taboos have an impact on their decision-making process regarding FGCS, making them more inclined to consider FGCS. However, most participants stated that this information does not influence their decision to undergo FGCS. The most reported source of information included social media (36.2%), online search (14.8%), friends or family (11.5%), while 30.4% had no information about FGSC. Generally, most of the participants (78.3%) had poor knowledge about FGCS.

**Table 3:** Women's knowledge of genital cosmetic surgery in Saudi Arabia FGCS - female genital cosmetic surgery

Knowledge items	No	%
Have you heard of female genital cosmetic surgery (FGCS)?		
Yes	291	49.4%
No	298	50.6%
What do you know about FGCS?		
It involves surgical procedures to change the appearance of the female genitalia	165	28.0%
It is also known as vaginal rejuvenation or designer vagina surgery	144	24.4%
It can involve procedures such as labiaplasty, clitoral hood reduction, or vaginal tightening	200	34.0%
It is sometimes done for aesthetic reasons but may also be done for medical reasons	190	32.3%
I don't know	271	46.0%
Would you like to learn more about FGCS?		
Yes	316	53.7%
No	273	46.3%
How do you feel about the availability of information about FGCS in Saudi Arabia?		
There is enough information available	105	17.8%
There is not enough information available	265	45.0%
I am not sure	219	37.2%
How do these information or taboos influence your attitudes toward FGCS?		
They make me more likely to consider FGCS	131	22.2%
They make me less likely to consider FGCS	96	16.3%
They do not influence my decision to consider FGCS	362	61.5%
Source of information about FGCS		
Healthcare provider	42	7.1%
Social media	213	36.2%
Online search	87	14.8%
Friends or family	68	11.5%
No specific source	179	30.4%
Overall knowledge level		
Poor	461	78.3%
Good	128	21.7%

Socio-demographics of women who responded to the question that FGCS involves procedures such as labiaplasty, clitoral hood reduction, or vaginal tightening are presented in Table 4. The sample was composed of 41.4% of the participants aged between 18 and 24 years compared to 18.8% of participants aged 55 years or older (p=0.002). Additionally, 42% of them were single, while 30.7% were married participants (p=0.043). Likewise, 60% of women with cardiac disease indicated that FGCS can involve procedures such as labiaplasty, clitoral hood reduction, or vaginal tightening in comparison to 32.5% of those without chronic disease (p=0.001). 

**Table 4:** Socio-demographics of women who noted that FGCS can involve procedures such as labiaplasty, clitoral hood reduction, or vaginal tightening Pearson X2 test; * p<0.05 is considered significant FGCS - female genital cosmetic surgery

Socio-demographics	FGCS involves procedures such as labiaplasty, clitoral hood reduction, or vaginal tightening				p-value
	Yes		No		
	#	%	#	%	
Age in years					0.002*
18-24	63	41.4%	89	58.6%	
25-34	88	38.6%	140	61.4%	
35-44	31	25.0%	93	75.0%	
45-54	15	21.7%	54	78.3%	
55+	3	18.8%	13	81.3%	
Marital status					0.043*
Single	60	42.0%	83	58.0%	
Married	123	30.7%	278	69.3%	
Divorced / widow	17	37.8%	28	62.2%	
Educational level					0.065
Below secondary	4	17.4%	19	82.6%	
Secondary / diploma	41	27.7%	107	72.3%	
Bachelor degree	136	37.2%	230	62.8%	
Post-graduate	19	36.5%	33	63.5%	
Employment					0.803
Housewife	82	31.9%	175	68.1%	
Student	36	38.7%	57	61.3%	
Employed full-time	59	33.5%	117	66.5%	
Employed part-time	14	35.9%	25	64.1%	
Self-employed	9	37.5%	15	62.5%	
Monthly income					0.085
<5000 SR	124	37.5%	207	62.5%	
5000-10000 SR	44	33.8%	86	66.2%	
10000-15000 SR	15	20.3%	59	79.7%	
15000-20000 SR	11	32.4%	23	67.6%	
>20000 SR	6	30.0%	14	70.0%	
Chronic diseases					0.001*
None	138	32.5%	286	67.5%	
Diabetes	24	42.1%	33	57.9%	
Hypertension	18	41.9%	25	58.1%	
Hypothyroidism	19	33.9%	37	66.1%	
Cardiac disease	9	60.0%	6	40.0%	
Asthma	25	53.2%	22	46.8%	

Women's practices of FGCS in Saudi Arabia are presented in Table 5. Only 45 (7.6%) underwent FGCS, but 112 (19%) confirmed they consider undergoing FGCS for themselves. The most considered were vaginal tightening (38.5%), labiaplasty (27.3%), and clitoral hood reduction (17.6%). The common factors behind this consideration were personal dissatisfaction with the appearance of genitalia (45.5%), partner's dissatisfaction with the appearance of genitalia (15%), and pain/discomfort during sexual activity (15%). A total of 109 (18.5%) participants talked about FGCS with a healthcare provider; 61.5% of them were supportive and provided information, 24.8% were neutral or did not provide much information, and 13.8% were dismissive or judgmental. 

**Table 5:** Women's practices of FGCS in Saudi Arabia FGCS - female genital cosmetic surgery

Practice	No	%
Have you ever considered undergoing FGCS yourself?		
Yes	112	19.0%
No	402	68.3%
May be	75	12.7%
If yes, what type of FGCS are you considering?		
Vaginal tightening	72	38.5%
Clitoral hood reduction	33	17.6%
Labiaplasty	51	27.3%
Others	31	16.6%
How did you come to the decision to consider FGCS?		
Personal dissatisfaction with the appearance of genitalia	85	45.5%
Partner's dissatisfaction with the appearance of genitalia	28	15.0%
Pain/discomfort during sexual activity	28	15.0%
Others	46	24.6%
Have you ever had any type of cosmetic surgery?		
Yes, FGCS	45	7.6%
Yes, another type of cosmetic surgery	56	9.5%
Never	488	82.9%
If yes, how did your experience with cosmetic surgery impact your decision to consider FGCS?		
Positive experience made me more likely to consider FGCS	44	43.6%
Negative experience made me less likely to consider FGCS	26	25.7%
No impact, the decision to consider FGCS is unrelated to previous cosmetic surgery experience	31	30.7%
Have you ever talked about FGCS with a healthcare provider?		
Yes	109	18.5%
No	480	81.5%
If yes, how did the healthcare provider respond?		
They were supportive and provided information.	67	61.5%
They were neutral or did not provide much information	27	24.8%
They were dismissive or judgmental	15	13.8%

Women's attitudes regarding FGCS in Saudi Arabia are presented in Table 6. Out of all participants, 84.6% think that it's important to have access to support services, such as counseling or peer support, 80% indicated that it is important that healthcare providers in Saudi Arabia are knowledgeable about FGCS, 72.2% were concerned about the potential risks and complications of FGCS, 62.8% agreed that it's important to have the procedure performed in Saudi Arabia when considering FGCS, 43.6% think that it's important that husband/partner supports the decision to undergo FGCS, and 30.2% think that discussing FGCS with husband partner is comfortable.

**Table 6:** Women's attitudes regarding FGCS in Saudi Arabia FGCS - female genital cosmetic surgery

Attitudes	No	%
Discussing FGCS with husband/partner is comfortable		
Not at all comfortable	178	30.2%
Not very comfortable	52	8.8%
Not sure	181	30.7%
Somewhat comfortable	117	19.9%
Very comfortable	61	10.4%
It's important that husband/partner supports the decision to undergo FGCS		
Not at all important	106	18.0%
Not very important	61	10.4%
Not sure	165	28.0%
Somewhat important	129	21.9%
Very important	128	21.7%
Is it important to have the procedure performed in Saudi Arabia if you are considering FGCS?		
Not at all important	57	9.7%
Not very important	47	8.0%
Not sure	115	19.5%
Somewhat important	107	18.2%
Very important	263	44.7%
I am concerned about the potential risks and complications of FGCS		
Not at all concerned	74	12.6%
Not very concerned	90	15.3%
Somewhat concerned	215	36.5%
Very concerned	210	35.7%
Is it important to you that healthcare providers in Saudi Arabia are knowledgeable about FGCS?		
Not at all important	56	9.5%
Not very important	62	10.5%
Somewhat important	137	23.3%
Very important	334	56.7%
Is it important to have access to support services, such as counseling or peer support?		
Not at all important	45	7.6%
Not very important	46	7.8%
Somewhat important	115	19.5%
Very important	383	65.0%
Do you think FGCS should be covered by health insurance in Saudi Arabia?		
Yes	419	71.1%
No	170	28.9%

Factors associated with participants' knowledge about genital cosmetic surgery are presented in Table 7. A notable finding indicated that 25.7% of young women had a good understanding of FGCS, compared to 12.5% of women aged 55 years or older, and this difference was statistically significant (p=0.049). Also, 26.9% of highly educated women had a good knowledge level versus 8.7% of other participants with lower education (p=0.046). Good knowledge about FGCS was indicated among 43.8% of women who have ever considered undergoing FGCS compared to 16.4% of those who have not (p=0.001), and among 44.4% of other participants who had any type of cosmetic surgery versus 19.9% of those who hadn't (p=0.001). Likewise, 36.7% of women who talked about FGCS with a healthcare provider had a good knowledge level about the surgery versus 18.3% of others who didn't (p=0.001).

**Table 7:** Factors associated with participants' knowledge about FGCS Pearson X2 test; $ exact probability test; * p<0.05 is considered significant FGCS - female genital cosmetic surgery; NVD - normal vaginal delivery; CS - Cesarean section

Factors		Overall knowledge level				p-value
		Poor		Good		
		No	%	No	%	
Age in years	18-24	113	74.3%	39	25.7%	0.049*^$^
	25-34	172	75.4%	56	24.6%	
	35-44	100	80.6%	24	19.4%	
	45-54	62	89.9%	7	10.1%	
	55+	14	87.5%	2	12.5%	
Marital status	Single	109	76.2%	34	23.8%	0.323
	Married	320	79.8%	81	20.2%	
	Divorced / widow	32	71.1%	13	28.9%	
Educational level	Below secondary	21	91.3%	2	8.7%	0.046*
	Secondary / diploma	123	83.1%	25	16.9%	
	Bachelor degree	279	76.2%	87	23.8%	
	Post-graduate	38	73.1%	14	26.9%	
Monthly income	<5000 SR	257	77.6%	74	22.4%	0.623
	5000-10000 SR	99	76.2%	31	23.8%	
	10000-15000 SR	63	85.1%	11	14.9%	
	15000-20000 SR	27	79.4%	7	20.6%	
	>20000 SR	15	75.0%	5	25.0%	
Age at first sexual intercourse	Never had sexual intercourse	109	76.2%	34	23.8%	0.504
	<18	34	75.6%	11	24.4%	
	18-24	213	77.2%	63	22.8%	
	25-34	57	82.6%	12	17.4%	
	35+	48	85.7%	8	14.3%	
Frequency of sexual activity per week	Less than once a week	102	77.9%	29	22.1%	0.483^$^
	1-2 times a week	143	76.5%	44	23.5%	
	3-4 times a week	78	83.9%	15	16.1%	
	>4 times a week	29	82.9%	6	17.1%	
Mode of delivery	NVD	188	78.3%	52	21.7%	0.577
	CS	67	80.7%	16	19.3%	
	Instrumental delivery	14	70.0%	6	30.0%	
Have you experienced any complications during pregnancy or delivery?	Yes	65	80.2%	16	19.8%	0.701
	No	216	78.3%	60	21.7%	
Have you undergone perineal tear/episiotomy during the previous delivery?	Yes	104	73.2%	38	26.8%	0.123
	No	140	81.4%	32	18.6%	
	Not sure	25	86.2%	4	13.8%	
Source of information about FGCS	Healthcare provider	30	71.4%	12	28.6%	0.001*
	Social media	137	64.3%	76	35.7%	
	Online search	64	73.6%	23	26.4%	
	Friends or family	52	76.5%	16	23.5%	
	No specific source	178	99.4%	1	.6%	
Have you ever considered undergoing FGCS yourself?	Yes	63	56.3%	49	43.8%	0.001*
	No	336	83.6%	66	16.4%	
	May be	62	82.7%	13	17.3%	
Have you ever had any type of cosmetic surgery?	Yes, FGCS	25	55.6%	20	44.4%	0.001*
	Yes, another type of cosmetic surgery	45	80.4%	11	19.6%	
	Never	391	80.1%	97	19.9%	
Have you ever talked about FGCS with a healthcare provider?	Yes	69	63.3%	40	36.7%	0.001*
	No	392	81.7%	88	18.3%	

## Discussion

FGCS involves changing the appearance of the labia or vulva [12]. Some individuals select this surgery because they are dissatisfied with the appearance of their genitalia. This type of surgery is also referred to as "aesthetic genital surgery" [13]. Female genital reshaping, or labiaplasty, specifically focuses on changing the size and shape of the inner labia or the inner lips of the vulva. It is important to note that labiaplasty and phalloplasty are not the same as gender reassignment surgeries [14,15].

The current study aimed to assess females' attitudes and knowledge towards genital cosmetic surgeries in Saudi Arabia. Regarding knowledge level, the current study showed that about one-fifth of the women who participated in the study had good knowledge about the surgeries. Approximately half of the study participants were familiar with the term FGCS. The most common definition provided was as follows: "It can involve procedures such as labiaplasty, clitoral hood reduction, or vaginal tightening". Less than one-fifth of the study women think that there is enough information available about FGCS in Saudi Arabia, and about one-fifth indicated that information or taboos influence their decision toward FGCS, making them more likely to consider FGCS, but most of them noted that information does not influence their decision to consider FGCS. The most reported source of information included social media, which played the main role in providing participants with needed information about FGCS, which was similar to other studies' conclusions [16,10]. Other less frequent sources, including online searches, friends, or family, were reported. Similar findings were reported among medical students in Saudi Arabia by Iqbal et al. [17], where about 68.7% of the participants did not have much knowledge about vulvovaginal aesthetics procedures. Also, in India, Gowardhan and Bagade [18] documented that 26.2% of females had satisfactory knowledge about FGCS. Social media (56.5%) was the most common source of information for these participants. A much lower awareness level was reported by Bello and Lawal [19], as only 27.7% heard about FGCS. In contrast to the current study, medical personnel were the main source of information in their study. Regarding factors associated with women's knowledge, young age, high education, considering FGCS, undergoing cosmetic surgery, and talking about FGCS with a healthcare provider were significant predictors.

Regarding women's attitudes, the current study showed that most of them think that it's important to have access to support services, such as counseling or peer support, and it is important that healthcare providers in Saudi Arabia are knowledgeable about FGCS. About three-fourths were concerned about the potential risks and complications of FGCS, and about two-thirds agreed that it's important to have the procedure performed in Saudi Arabia when considering FGCS. Less than half think that it's important that the husband/partner supports the decision to undergo FGCS, and one-third think that discussing FGCS with the husband/partner is comfortable. All these indicate a nearly positive attitude towards the procedure with the need for support and availability of surgeries in Saudi Arabia.

Limitations that were faced while conducting this study were the difficulty of reaching participants from other regions as most of our team were from the central region; therefore, it affected the regional composition of participants who were reached. Another limitation is the unwillingness of the participants to take part in the survey due to the stigmatized views around the topic among the Saudi population. Finally, another obstacle was translating medical and technical terms related to our research into the Arabic language to make sure that our participants were able to fully understand the context and the scope of our research.

## Conclusions

In conclusion, the current study revealed that nearly one out of five women were knowledgeable about FGCS, mainly about the nature and types of the procedure. Higher knowledge was associated with young age, high education, and future consideration of undergoing the surgeries. Social media was the main player in gaining information, with no role for healthcare staff. Also, the females who participated in the study had a positive attitude towards FGCS, mainly if supported by partners and their healthcare staff with the availability of surgeries in Saudi Arabia. Saudi women should be informed mainly by healthcare staff in detail about the merits and hazards of the FGCS procedures to make a precise, informed decision about the FGCS procedures.
